# Engineered Drug Resistant γδ T Cells Kill Glioblastoma Cell Lines during a Chemotherapy Challenge: A Strategy for Combining Chemo- and Immunotherapy

**DOI:** 10.1371/journal.pone.0051805

**Published:** 2013-01-11

**Authors:** Lawrence S. Lamb, Joscelyn Bowersock, Anindya Dasgupta, G. Yancey Gillespie, Yun Su, Austin Johnson, H. Trent Spencer

**Affiliations:** 1 Department of Medicine, University of Alabama at Birmingham, Birmingham, Alabama, United States of America; 2 Department of Surgery, University of Alabama at Birmingham, Birmingham, Alabama, United States of America; 3 Emory University School of Medicine, Department of Pediatrics, Aflac Cancer Center and Blood Disorders Service, Atlanta, Georgia, United States of America; University of Michigan School of Medicine, United States of America

## Abstract

Classical approaches to immunotherapy that show promise in some malignancies have generally been disappointing when applied to high-grade brain tumors such as glioblastoma multiforme (GBM). We recently showed that *ex vivo* expanded/activated γδ T cells recognize NKG2D ligands expressed on malignant glioma and are cytotoxic to glioma cell lines and primary GBM explants. In addition, γδ T cells extend survival and slow tumor progression when administered to immunodeficient mice with intracranial human glioma xenografts. We now show that temozolomide (TMZ), a principal chemotherapeutic agent used to treat GBM, increases the expression of stress-associated NKG2D ligands on TMZ-resistant glioma cells, potentially rendering them vulnerable to γδ T cell recognition and lysis. TMZ is also highly toxic to γδ T cells, however, and to overcome this cytotoxic effect γδ T cells were genetically modified using a lentiviral vector encoding the DNA repair enzyme O(6)-alkylguanine DNA alkyltransferase (AGT) from the O(6)-methylguanine methyltransferase (MGMT) cDNA, which confers resistance to TMZ. Genetic modification of γδ T cells did not alter their phenotype or their cytotoxicity against GBM target cells. Importantly, gene modified γδ T cells showed greater cytotoxicity to two TMZ resistant GBM cell lines, U373^TMZ-R^ and SNB-19^TMZ-R^ cells, in the presence of TMZ than unmodified cells, suggesting that TMZ exposed more receptors for γδ T cell-targeted lysis. Therefore, TMZ resistant γδ T cells can be generated without impairing their anti-tumor functions in the presence of high concentrations of TMZ. These results provide a mechanistic basis for combining chemotherapy and γδ T cell-based drug resistant cellular immunotherapy to treat GBM.

## Introduction

Treatment strategies for high-grade primary brain tumors such as glioblastoma multiforme (GBM) have failed to significantly and consistently extended survival despite 50 years of advances in radiotherapy, chemotherapy, and surgical techniques [1]. Immunotherapy remains an attractive option, although classical approaches that have shown some promise in other malignancies have generally been disappointing when applied to GBM [2]–[7]. A variety of immune cell therapy approaches to GBM have been attempted over the past several years. *Ex vivo* culture of cytotoxic T lymphocytes (CTL) from tumor-draining lymph nodes [8], [9], tumor-infiltrating lymphocytes (TIL), and HLA-mismatched T cells from healthy donors with systemic and intracranial infusion have all met with limited success. The most predominant cell therapy consisted of autologous lymphokine-activated killer (LAK) cells, a combination of NK and T lymphocytes cultured in high doses of IL-2. Although promising in early studies, these therapies fall short for several reasons. CTL therapies are based on adaptive immunity (i.e. MHC-restricted, antigen-specific responses) and are therefore dependent upon the dose of T cell clones that specifically recognize various tumor-associated peptide antigens dispersed among various subsets of glioma cells. Infusion or intracranial placement of HLA-mismatched CTL relies on allogeneic recognition of transplantation antigens and is highly dependent on glioma cell MHC Class I expression [10], [11]. LAK cell preparations are difficult to consistently manufacture, are short-lived *in vivo* [12], and are complicated by IL-2 related toxicity once infused or placed in the tumor resection cavity [2], [13]–[16].

To overcome these issues, during the past six years, we developed a robust method for generating anti-glioma immunocompetent γδ T cells. We have shown that *ex vivo* expanded/activated γδ T cells from healthy volunteers are cytotoxic to high-grade gliomas in both *in vitro* and in specific *in vivo* models designed to replicate therapeutic conditions [17]–[19]. The anti-tumor cytotoxicity of γδ T cells is at least partially due to innate recognition of stress-induced NKG2D ligands such as MICA/B and UL-16 binding proteins (ULBP) that are expressed on GBM but not on adjacent normal brain tissue [17], [20], [21].

One of the most formidable obstacles in the treatment of cancer has been chemotherapy-induced hematopoietic cell toxicity and the associated loss of an effective and robust immune response [22]. To circumvent these consequences, concurrent with the development of immunocompetent cell expansion methods, we developed a gene therapy-based strategy whereby anti-cancer immune cells are genetically engineered to resist the toxic effects of chemotherapy drugs, which allows for the combined administration of chemotherapy and immunotherapy. This drug resistant immunotherapy (or DRI) approach has been shown to be effective in animal models of sarcoma and neuroblastoma. [23]–[25].

Temozolomide (TMZ) - induced DNA damage induces transient expression of NKG2D ligands on cells that are generally resistant to the drug, rendering them vulnerable to recognition and lysis by γδ T cells [26]. Strategies that protect cellular therapy products from chemotherapy induced toxicity could likely improve the effectiveness of combined immune and chemotherapy regimens. In this report, an *in vitro* proof of concept evaluation of a DRI-based strategy using lentiviral genetic modification of γδ T cells for enforced expression of P140KMGMT, which confers resistance to TMZ, is presented as a previously unexplored avenue for treatment of high-grade gliomas.

## Methods

Blood samples were obtained from consenting volunteers, in writing, in accordance with the principles expressed in the Declaration of Helsinki and was approved by the University of Alabama at Birmingham's Institutional Review Board.

### Glioblastoma cell lines and cloning of TMZ-resistant cells

Human glioma cell lines U87, U373, and SNB-19 were used in this study. The U87 is a grade IV glioma that originated from a 44-year-old Caucasian woman [27]. The genetic characteristics of the cell line have been well-described [28]. The cell line was obtained from the ATCC by the UAB Brain Tumor Tissue Core, a unit of the UAB NCI SPORE in Brain Cancer. Its origin has been verified by STR PCR and has been found to agree with the original cell source. U373MG is a grade III astrocytoma that was cultured from a 61 year old male [27] and was obtained directly from Darell D. Bigner (Duke University) who obtained them from Jan Ponten of Uppsala University. The cell line has been verified as authentic (Rb-deleted, p15/p16 wildtype) and has the same STR pattern as the original line. SNB-19 is a grade IV glioma cell line derived from the resection of a glioblastoma multiforme from a 47 year old male [29] and was obtained directly from Richard Morrison who extensively characterized the cell line [30]. The cells have been verified as authentic by STR PCR.U87 cells, known to be resistant to TMZ, were not modified. SNB-19 and U373 glioma cells, normally sensitive to TMZ, were cultured in incremental concentrations of TMZ up to 400 µM over several weeks in our laboratory with stepwise selection and subculture of resistant clones as described by Zhang [31].

### Expansion and activation of human γδ T Cells

Peripheral blood (50 ml) was obtained from healthy volunteers. Mononuclear cells were isolated by density gradient centrifugation and resuspended at 1.0×10^6^/ml in RPMI 1640+10% autologous serum +1 µM Zoledronic Acid (Novartis Oncology; East Hanover, NJ) with 50 U/ml IL-2 (Chiron; Emeryville, CA). Cells were transduced with lentivirus on culture day +6 and +7 as described below, and the culture was maintained at the original density for 14 days with addition of 50 U/ml IL-2 on post-culture days 2, 6, and 10 and addition of complete media as determined by pH and cell density. Composition, purity, and viability were determined by flow cytometry at day 0, +7 and +14 following initiation of the culture. A final viability determination was obtained by flow cytometric analysis of ToPro Iodide (Molecular Probes; Eugene, OR) incorporation. Our final product routinely contains ≥80% γδ T cells, ≤5% αβ T cells, and ≤15% NK cells to be acceptable for further studies.

### Lentivirus vector production and titer

The SIV vector used in the proposed studies is based on the SIVmac viral system obtained from Dr. Arthur Nienhius (St. Jude Children's Hospital, Memphis, TN) and has been described previously [24], [32], [33]. Transgenes that confer drug resistance were cloned into the SIV transfer vector, pCL20cSLFR MSCVGFP, between the BstEII and Not1 restriction sites. A CMV promoter was then cloned in place of the MSCV promoter to generate pCL20-CMV-P140KMGMT. The control vector, pCL20-CMV-GFP, contains GFP driven by the CMV promoter. All recombinant viral-based vectors were prepared by transient co-transfection of 293T cells with the following plasmids: pSIV:2.06 µg, PCAG4:1.25 µg, pVSVG:1.25 µg, pCL20 expression vector:1.67 µg using 40 µl of Lipofectamine 2000 per 10 cm plate. One day post-transfection, the media was replaced with fresh DMEM-F12, 10% FBS, 1% penicillin/streptomycin and viral supernatant was collected every 24 hours for three days. Pooled viral supernatant was filtered through a 0.45 µM filter and concentrated overnight by sedimentation at 10,000× *g*. The pellet was resuspended in StemPro 34 media at 1/100^th^ the initial volume and frozen in 1 ml aliquots. The concentrated vector was titered by transducing HEK-293T cells with increasing vector volumes. Seventy two hours post-transduction genomic DNA was isolated and DNA copy number was estimated by quantitative PCR. Titers were determined using primers designed specifically to amplify the transgene. Typically, virus titers of 10^8^ TU/ml were obtained.

### Lentiviral transduction of γδ T cells

On the previously described days of expansion culture, 1×10^6^ γδ T cells were added to 1 ml of pre-warmed culture medium and plated in a 6 well culture dish. To each well varying amounts of virus were added to achieve an MOI of 5, 10, 20, and 50, with 2 control wells, as well as 50 U of IL-2. For 3 consecutive days, beginning on day 6, viable cell counts were performed and virus was added to the media to obtain the desired MOI. Additional media was added to each well to bring the total volume to 1 mL if needed. On day 9, 11, 13 and 15 a viable cell count was performed and media added to bring the concentration of viable cells to 1×10^6^ cells/mL; with additional IL-2 added to a concentration of 50 U/mL On day 15 the cells were incubated in media containing 400 µM TMZ for 24 hours. Following incubation viable cell counts were measured using an automated Trypan-blue dye exclusion and counting system(Vi-Cell: Beckman-Coulter; Miami, FL).

To prepare a bulk γδ T cell culture, lentivirus was added to the cell culture medium at an MOI of 20 and supplemented with 6 µg/ml polybrene. The transduction was repeated the following day with additional lentivirus particles, also at an MOI of 20. Twenty four hours after the second transduction, fresh medium was added to the virus containing medium and the transduced cells were used within a week of preparation.

### Flow cytometry and NKG2DL assays

Cultured peripheral lymphocytes were labeled with fluorochrome-conjugated antibodies to CD3 (SK7) and TCR-γδ (11F2) (BD Biosciences: San Jose, CA). For NKG2DL assays, SNB-19, U373, and U87MG human glioma cells were cultured as described below in equal volumes of DMEM-F12 and HAM's media with 10%FCS supplemented with 2 mM l-glutamine until confluent. Cells were removed, washed in PBS, and resuspended in PBS containing 5% FBS and 100 µM aqueous TMZ (control cells received PBS only) and labeled with NKG2D ligands MIC-A/B conjugated with Phychoerythrin (PE), ULBP-1 PE, ULBP-2 conjugated wit Allophycocyanin (APC), ULBP-3 PE, ULBP-4 PE, and appropriately matched isotype controls (R&D Systems; Minneapolis, MN) for 20 min at 4°C. Following a second wash, the cells were acquired on a BD FACS Canto Flow Cytometer at intervals of 1,2,4,8, and 24 hours. Minimums of 10,000 events were acquired and analyzed using FACS DiVa and CellQuest Pro software (BD Biosciences; San Jose, CA). Median Fluorescence Intensity (MFI) was calculated from individual histograms and expressed as MFI ± SD of each curve. Single tubes were acquired for each experiment and separate duplicate experiments were performed to verify trends.

### Cloning of TMZ Resistant Cell Lines

TMZ-resistant cells were cloned as described by Zhang [31]. SNB19 and U373 cell lines were cultured in six-well polypropylene plates in equal volumes of DMEM-F12 and HAM's media. Starting with 1 µM, cells were cultured in incrementally increasing TMZ concentrations of 1, 2, 5, 10, 20, 50 and finally 100 µM until cells could be passaged in 100 µM TMZ. The procedure required approximately six months to achieve small numbers of replicating TMZ-resistant cells that are highly resistant to TMZ and show strong expression of NKG2DL ULBP-2 and ULBP-3.

### Cytotoxicity assays

Potency of the cell product was determined using *in vitro* cytotoxicity assays against the unmodified and TMZ-resistant clones of the SNB-19 and U373 cell lines and normal astrocyte cultures (control for toxicity). Targets were labeled with the membrane dye PKH26 (Sigma; St. Louis, MO). Expanded/activated γδ T cells were then added to the tubes at ratios of 0∶1 (Background), 5∶1, 10∶1, 20∶1 and 40∶1 effectors/GBM targets, incubated for four hours at 37°C and 5% CO_2_, washed once and resuspended in 1 ml HBSS. ToPro Iodide solution (20 µl) (Molecular Probes; Eugene, OR) was added prior to acquisition on the flow cytometer. Cytotoxicity was calculated as: (Toprolodide^+^PKH26^+^ events/total PKH26^+^ events) ×100. Single tubes were acquired for each experiment and duplicate experiments were performed as quality control.

### Statistical analysis

Descriptive statistics were used to characterize mean, standard deviation, and standard error of populations. For comparison of antigen expression, median fluorescence intensity (MFI) was obtained from single parameter histograms. Comparisons between groups was accomplished by single parameter t-test for differences between means and the Wilcoxon Signed-Rank test for differences in medians. A result was considered significant at a *p* value of 0.05.

## Results

### NKG2D Ligands are transiently upregulated on TMZ-resistant U87MG glioma cells after exposure to TMZ

**Figure 1:**
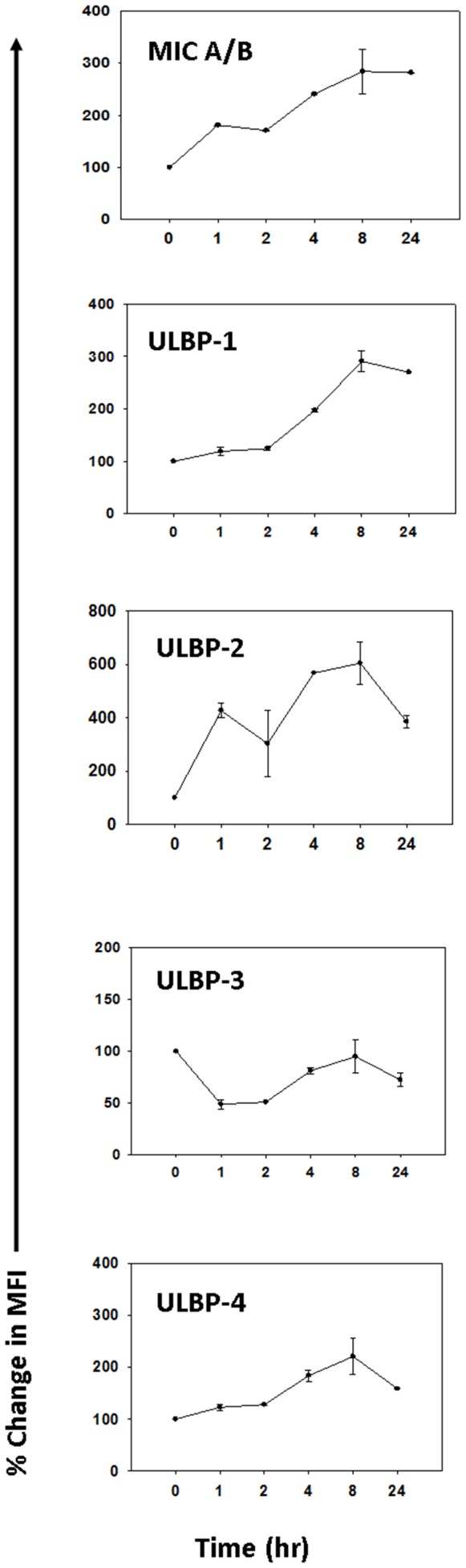
Transitory increase in stress-associated antigens on TMZ-resistant cell line U87 after exposure to TMZ. U87 cells were cultured to confluence and incubated in media containing 100 µM TMZ. Stress antigens were assessed at the time intervals noted on the x axis by flow cytometry and the increase in median fluorescence intensity over isotype control were calculated. Data are shown as percentage increase over unmanipulated U87 cells. SD of 3 experiments are shown.

In this experiment, we sought to determine if TMZ exposure stresses a TMZ-resistant U87 culture that had not previously been exposed to TMZ, NKG2D ligand expression was examined at incremental time intervals following TMZ exposure. Chemotherapy-induced stress was observed as demonstrated by transient up-regulation of the NKG2D ligands ULBP-1, -2, -4, and MIC A/B over the first several hours following exposure (**Figure 1**). In most cases, upregulated surface expression of NKG2DL began to normalize within 24 hours. These results indicate that the increase in NKG2D ligand expression in response to TMZ could increase the vulnerability of glioma cells to recognition and lysis by γδ T cells within the first 4 to 6 hours following TMZ-based chemotherapy.

### Generation of TMZ-resistant γδ T cells

**Figure 2:**
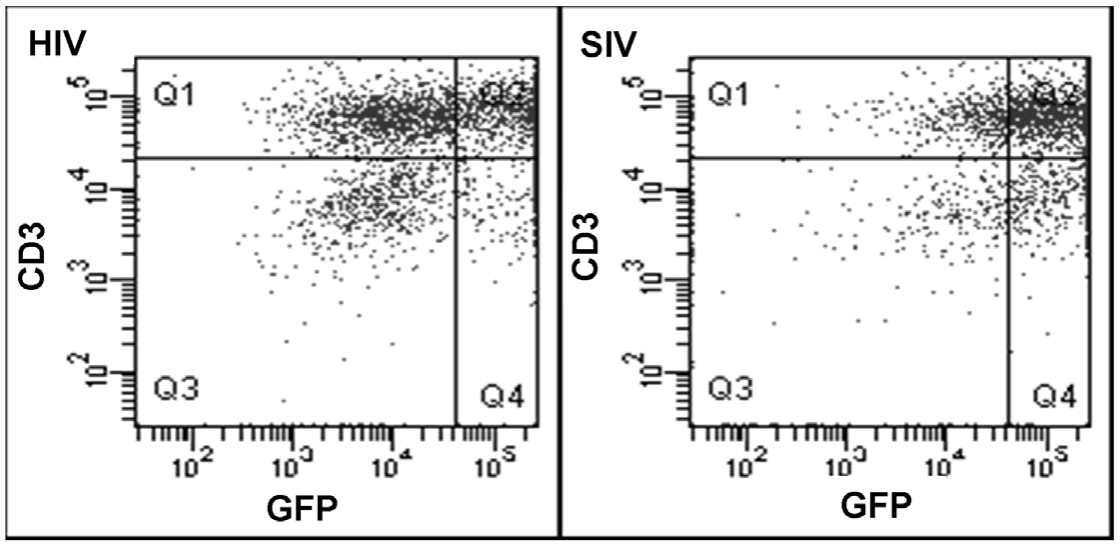
Genetic modification of expanded/activated γδ T cells by HIV (a) and SIV-GFP (b) lentiviral vectors 6 days following transduction with an MOI of 15. Mean fluorescence intensity (MFI) is approximately the same for both vectors, but transduction efficiency and expression from the SIV-derived vector is higher when measured on day+6. Quadrant values are noted to the right of each plot.

To produce expanded/activated γδ T cells that retain function when exposed to high concentrations of TMZ chemotherapy, vectors were generated that confer TMZ-resistance based on enforced expression of AGT. SIV- and HIV- based lentiviral vectors were initially compared to optimize the transduction efficiency of γδ T cells. Using the 14 day expansion culture described above, γδ T cells were transduced at an initial MOI of 15 with HIV-GFP or SIV-GFP vectors on days 6, 7, and 8. Transgene expression was assessed using flow cytometry. As shown in ***Figure 2***, the SIV-based vector transduced γδ T cells with a higher efficiency (Q2 = 65%) compared to an HIV-based vector (Q2 = 42%) (*n* = 3, *p* = 0.04).

**Figure 3:**
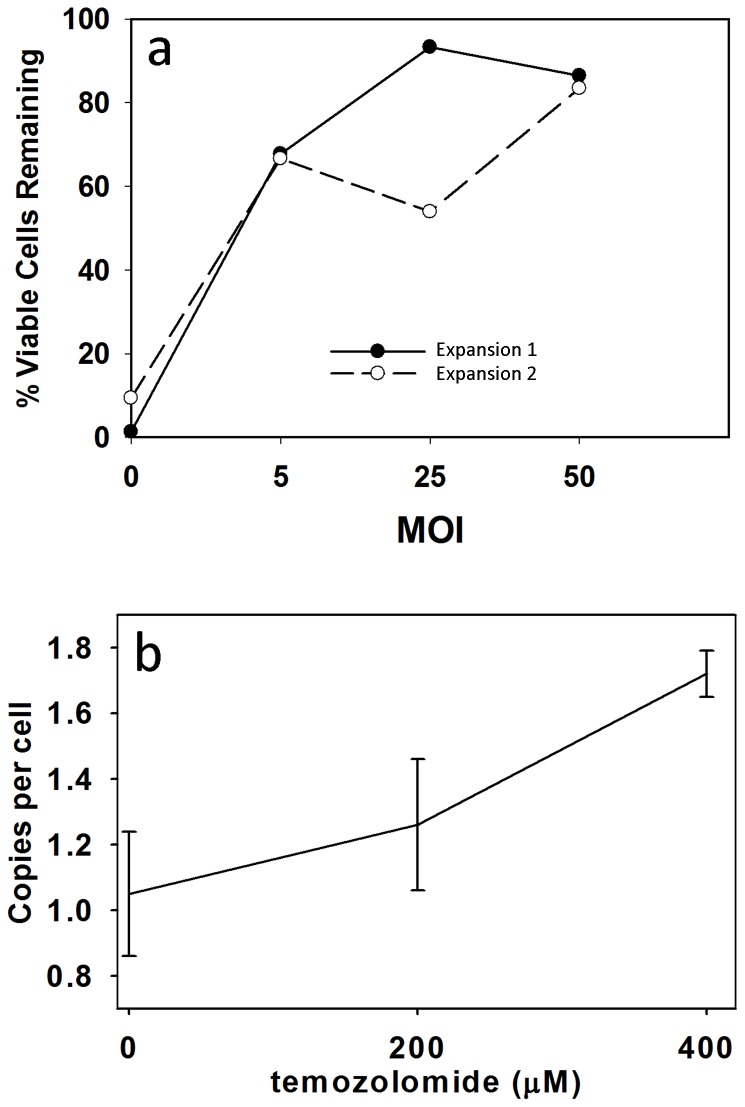
Transduction of γδ T cells with lentivirus vector was performed on day 6, 7 and 8 of expansion culture (see text) with increasing MOI. (a) On day +14 cells were incubated in media supplemented with 400 µM TMZ and viable cell counts were obtained for each MOI. Two separate experiments are shown. (b) Quantitative PCR analysis to measure P140KMGMT copy numbers of the bioengineered γδ T cells in the presence of increasing concentrations of TMZ, which are indicated in the figure.

An SIV-based vector expressing the MGMT transgene was then tested from an MOI of 5 to 50 at cell concentrations of approximately 3.5×10^3^ cells/µL (range 2.9–4.2×10^3^) to determine the ability of SIV-based vectors to modify and protect γδ T cells from TMZ-induced cytotoxicity. Following a three day transduction protocol and 14 days of culture, expanded/activated γδ T cells were incubated in media containing 400 µM TMZ for 24 h. Control γδ T cells not transduced with vector were virtually 100% non-viable when TMZ was added, but MGMT transduced cells were TMZ resistant as demonstrated by cell viability at each MOI tested (**Figure 3A**). The γδ T cells were then transduced at an MOI of 15 and cultured in 0, 200 or 400 µM TMZ. Copy number determined by quantitative PCR increased with increasing TMZ concentrations, likely due to the selection of cells expressing greater amounts of MGMT *(* ***Figure 3B*** *)*.

### Genetic engineering of γδ T cells does not alter their response to Zoledronic acid/IL-2 expansion or cytotoxic function

**Figure 4:**
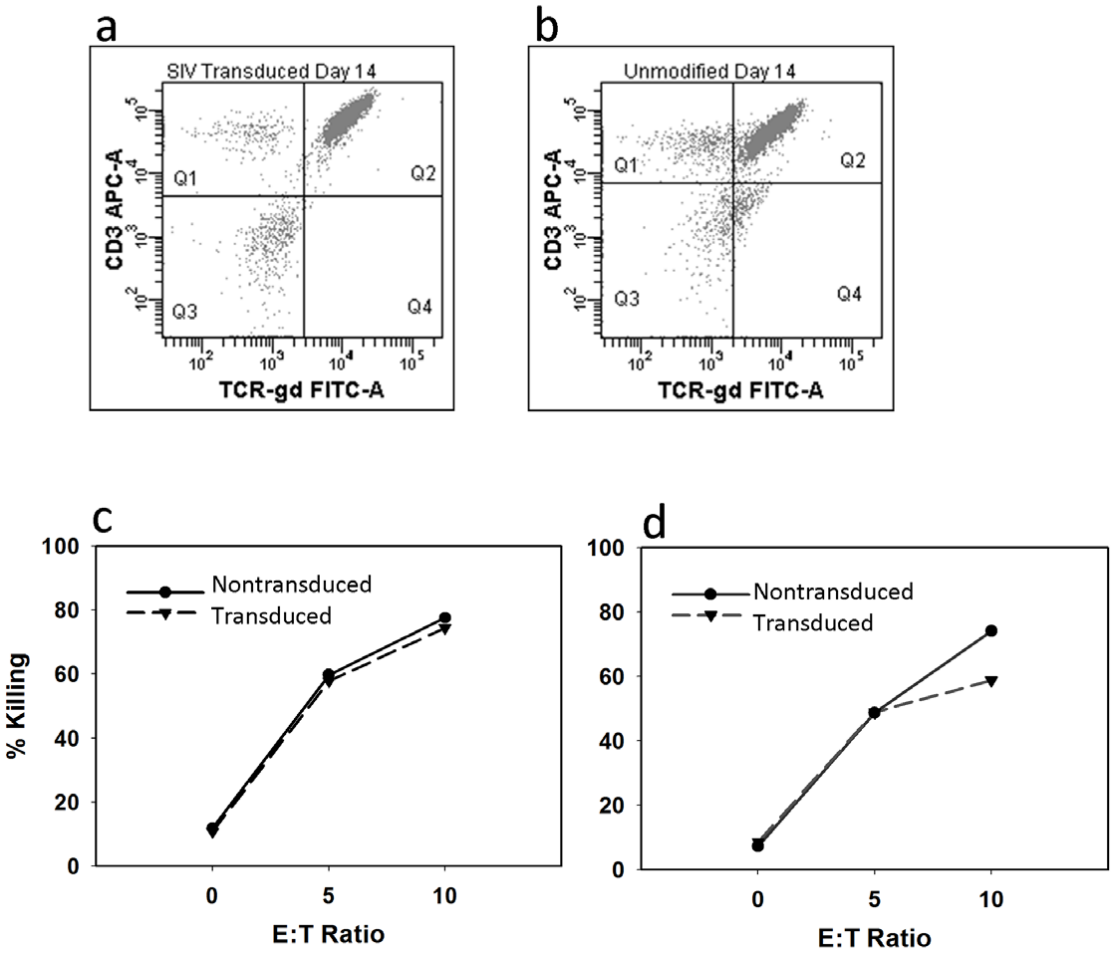
Expanded/activated γδ T cells were manufactured as described in the text. Flow cytometry from two separate donors shown from (*a*) unmanipulated and (*b*) P140KMGMT-transduced γδ T cells. For both panels (a) and (b) quadrant 2 (Q2) represents γδ T cells. As discussed in the text, the yield of γδ T cells was slightly lower than control due to loss of cells during the transduction procedure; however, purity of the final product was not affected as both products from a single donor show >90% purity of γδ T cells. (c and d) Cytotoxicity assays from two separate expansions (panel c and d, respectively) of unmodified γδ T cells (solid line) versus TMZ P140KMGMT transduced γδ T cells (dashed line) against the TMZ-resistant glioma cell line U87 were conducted to determine if genetic modification impairs γδ T cell function. Cytolytic activity of γδ T cells against U87 cells was nearly equivalent at all E:T ratios, verifying that P140KMGMT transduced γδ T cells function is equivalent to that of unmodified γδ T cells.

We then tested whether genetic modification with the MGMT vector had an effect on the the proliferative or cytotoxic function of γδ T cells (TMZ-transduced/resistant T cells - γδ^TMZ-R^) in response to the Zoledronic acid and IL-2 expansion protocol. Two representative experiments using expanded/activated γδ T cells from separate donors are shown. When comparing genetically-modified γδ T cells to unmodified cells we found no difference in the proliferative response, as all populations routinely yielded an expansion of γδ T cells comprising 65% - 90% of the total lymphocyte population *(* ***Figure 4a and 4b*** *)*. The cytotoxicity of unmodified to modified γδ T cells to the U87 glioma cell line was nearly equivalent at all E:T ratios (***Figure 4c and 4d*** *)*, verifying that γδ^TMZ-R^ genetically-modified T cell function is equivalent to that of unmodified γδ T cells.

For three separate donors, the expanded cells comprised approximately 2.0–12.0×10^8^ cells with transduced cell number yields generally less than unmodified cells due to cell loss during lentivirus transduction *(* ***Table 1*** *)*. However, a 50 ml blood draw routinely yielded ≥2×10^8^ transduced γδ T cells, which is sufficient for a therapeutic intracranial cell dose.

**Table 1:** Proliferation of Modified vs. Transduced γδ T cells in Culture.

Specimen	Initial γδ T cell number	Final* (unmodified)	Fold Expansion	Final* (transduced)	Fold Expansion
20100504	5.1×10^6^	2.3×10^8^	46.3	2.0×10^8^	39.9
20100812	3.4×10^6^	1.6×10^8^	73.1	2.5×10^8^	46.1
20110308	2.8×10^6^	1.2×10^9^	438.5	5.4×10^8^	191.4

* Cell dose is extrapolated to final volume of unmodified cells based on starting volume removed for transfection.

### Gene-modified γδ T cells function in the presence of TMZ

**Figure 5:**
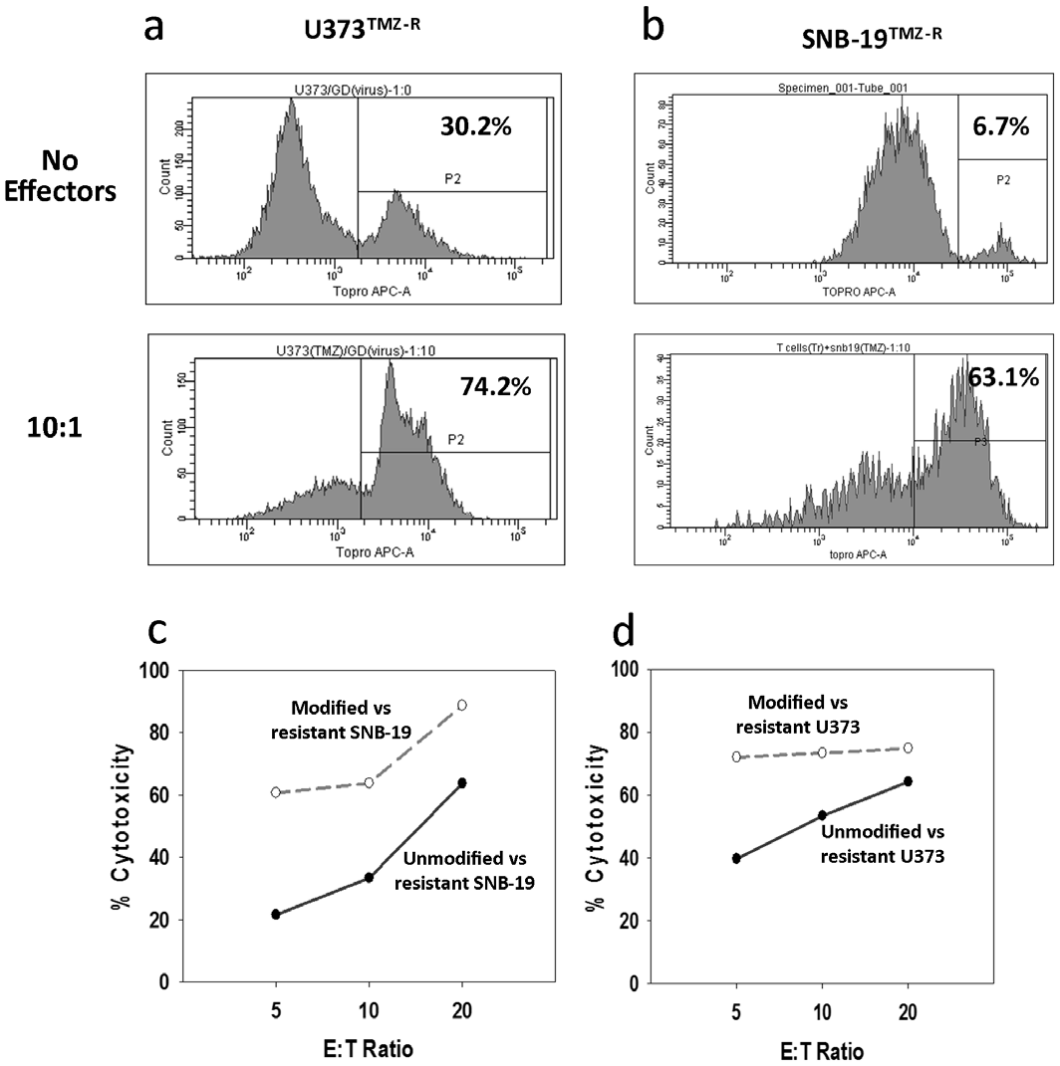
TMZ-resistant clones of the GBM cell line (*a*) U373^TMZ-R^ and (*b*) SNB-19^TMZ-R^ were selected by incubation in increasing concentrations of TMZ over 60 days. The cell lines were labeled with PKH-26 and incubated for 4 hours in the presence of 100 µM TMZ alone (upper panel) and with P140KMGMT transduced γδ T cells (γδ^TMZ-R^) at a 10∶1 effector:target ratio. The culture was then labeled with ToPro Iodide and acquired for flow cytometric phenotyping. A minimum of 5000 PKH26+ events was acquired to insure statistical validity of the data. All plots gated on PKH-26+ target cells. Note that the cloned cell lines are resistant to killing in media supplemented with TMZ with SNB-19^TMZ-R^ showing less cell loss than U373^TMZ-R^. Addition of γδ^TMZ-R^ results in much greater incorporation of ToPro Iodide after 4 h incubation suggesting that the increased cytotoxicity is overwhelmingly due to genetically modified γδ T cells. Dose-dependent cytotoxicity of γδ^TMZ-R^ is significantly less when assayed against SNB-19^TMZ-R^ (c) with no TMZ in the media vs. γδ^TMZ-R^ against SNB-19^TMZ-R^ in the presence of TMZ (p = 0.0085). Cytotoxicity was also trended greater against TMZ-resistant U373 with γδ^TMZ-R^ as effectors as well when the assay was conducted in the presence of TMZ (p = .0875). These assays were conducted as separate experiments from different donors.

Cytolytic function of MGMT-modified γδ T cells was evaluated against TMZ-resistant clones of SNB-19 and U373 in the presence of TMZ using the cytotoxicity assay procedure described above but modified to include 100 µM TMZ during the four-hour incubation. TMZ-resistant clones from both cell lines propagated slowly in TMZ-supplemented media but were highly resistant to the drug. As proof-of-concept, cytotoxicity against SNB-19^TMZ-R^ cells was assessed in separate experiments from U373^TMZ-R^ cells using different donors in order to conserve available cells while conducting the experiment in such a manner as to determine if expanded/activated γδ^TMZ-R^ function was consistent across donors and cell lines. TMZ-resistant clones remained viable in the absence of TMZ for the length of the assay as shown in the upper panels of Figure 5*a and 5b*. When both cell lines were incubated in the presence of TMZ and expanded/activated γδ^TMZ-R^, viability as measured by the uptake of the dye ToPro Iodide was noticeably increased after four hours in culture, as shown in the lower panels of Figure 5*a and 5b*. Dose-dependent cytotoxicity of WT γδ was significantly less when assayed against SNB-19^TMZ-R^ (c) with no TMZ in the media vs. γδ^TMZ-R^ against SNB-19^TMZ-R^ in the presence of TMZ (p = 0.0085). Cytotoxicity was also trended greater against TMZ-resistant U373 with γδ^TMZ-R^ as effectors as well when the assay was conducted in the presence of TMZ (p = .0875) but did not achieve significance at the p = 0.05 level. These assays were conducted as separate experiments from different donors.

## Discussion

No treatment options are currently available to control the progression of rapidly proliferating invasive high-grade gliomas. Intensive chemotherapeutic strategies, such as high dose temozolomide can lead to lymphodepletion, impaired T cell function, and consequent suppression of anti-tumor immune responses [34]. We have previously shown that local placement of allogeneic γδ T cells can slow progression of small established intracranial tumors and significantly extend survival in a human GBM xenograft model [35]. The characteristically rapid growth of high-grade gliomas as well as both systemic and local immunosuppression, however, remain formidable barriers to cellular therapy.

Several recent studies on solid extra-cranial neoplasms have shown that strategic timing of chemotherapy and immunotherapy, taking advantage of innate response to chemotherapy-induced expression of stress-associated antigens on tumor cells and depletion of regulatory T cells in the local microenvironment, can achieve synergies that are significantly greater than either individual approach [36]–[42]. It is envisioned that novel approaches to combine either traditional or new chemo- and immunotherapies can potentially improve upon the conventional treatment modalities for GBM.

Our DRI strategy presents an attractive avenue to effectively partner both immuno- and chemo- therapies by genetically engineering anti-cancer immune cells to confer anti-tumor immunity during aggressive dosing of chemotherapy. We recently showed that cultured leukemia cells can be eliminated by the combined additions of chemotherapy and genetically engineered immune effector cells [24]. We also showed that systemic administration of bioengineered chemotherapy-resistant hematopoietic cells has shown promise in animal models [23]. However, in the context of GBM therapy, systemic cell therapy will likely be an ineffective DRI strategy for established tumors due to their highly immunosuppressive nature of the tumor and the difficulty of the immune cells to cross the blood-brain barrier. However, systemic therapies incorporating DRI may be useful when directed at microscopic post-resection GBM. In the present study, we evaluated the effectiveness of a DRI strategy to enhance GBM cell clearance by the combined additions of genetically engineered γδ T cells with temozolomide to tumor cells that are refractory to high concentrations of the drug. Our choice to test a γδ T cell mediated DRI strategy is based upon our previous finding that γδ T cells, injected stereotactically either during intracranial transplantation or a few days after the transplantation of GBM cells in mice can extend the survival of the treated animals when compared to the survival of the tumor bearing animals that were not treated [35]. The exploitation of a γδ T cell based DRI strategy to target GBM is a practical approach since the tumor is partially shielded from the immune system, thereby preventing the elucidation of an immune response against locally infused cells.

A γδ T cell based DRI strategy against GBM cells can provide several benefits compared to chemotherapy alone, as cytotoxic drugs can potentially augment the cytolytic properties of the expanded γδ T cells. These cells express activating receptors for NKG2D family of ligands, such as ULBPs and MIC A/B, which are generally upregulated on stressed tumor cells. It has been established that tumors that express NKG2D ligands can readily be killed by immune effector cells that contain recognition receptors for these ligands [43], [44]. Such tumors are also often rejected during transplantation [45], while tumorigenesis is favored in mice that lack the expression of NKG2D receptors [46]. Surprisingly, in GBM cells, the efficacy of NKG2D mediated tumor destruction may be decreased in part due to elevated expression of MHC class I molecules on their surface [47]. However, tumor cell killing can be enhanced by forced expression of NKG2D ligands in GBM tumors [48]. We showed that the addition of temozolomide to drug resistant GBM cells induces transient but consistent upregulation of several NKG2D ligands on the U87 GBM cell line that displays partial resistance to TMZ. In this scenario, the addition of genetically engineered variants of the parental γδ T cells, that possess MHC unrestricted cytolytic properties, can potentially enhance tumor cell killing. The strategy of up-regulation of the stress/danger response of malignant cells following chemotherapy as a means of increasing their vulnerability to immune recognition and attack has been recently reviewed by others [26], [49], [50]. Consequently, up-regulation of stress-induced expression of NKG2D ligands on gliomas during chemotherapy can potentiate a DRI based anti-tumor strategy provided that immunocompetent cell therapies maintain efficacy during cytoreductive therapy.

We have also shown that in the presence of high concentrations of temozolomide the genetically engineered γδ T cells mediate significant killing of GBM cells that have been rendered resistant to temozolomide, whereas non-modified cells are ineffective. SNB-19 and U373 cell lines constitutively express high levels of surface NKD2D ligands ULBP-2 and ULBP-3 (data not shown) as well as MIC-A for U373, suggesting that the additive effect of TMZ on γδ T cell-based cytotoxicity may be partially mediated by nonpeptide ligands [51]. Besides inducing tumor associated stress molecules, chemotherapy can also augment immunotherapy in several ways, such as by enhancing the persistence of tumor reactive T lymphocytes and by increasing tumor trafficking of tumor responsive T cells, and by modulating immunosuppressive factors [52]. Thus administration of chemotherapy prior to cellular immunotherapy can modulate an immune environment that can be beneficial to the infused immune effector cells, such as γδ T cells. It has been shown that chemotherapy treatments can facilitate the rapid infiltration of large numbers of γδ T cells into tumors and prior to invasion of Tc1 cells [53]. Furthermore, temozolomide based chemotherapy has been shown to decrease the population of Fox-P3+ regulatory T cells, which provides an environment to further enhance the immune response [54].

Therefore, rapidly emerging evidence supports the crucial contribution of the innate immune system to the anti-tumorigenicity of conventional chemotherapy-based cancer treatments [22], [26], [49]. In the context of GBM therapy, in order to access the chemotherapy derived window of opportunity of tumor vulnerability it may be beneficial to place a high concentration of γδ T cells at the tumor site and to protect these effector cells, by gene transfer of MGMT, from the cytotoxic effects of TMZ chemotherapy, which would otherwise reduce or abrogate their function. In the present study, we successfully demonstrated two key aspects that are essential to the success of such a localized and a passive immunotherapy approach to target GBM: i) the genetic engineering of γδ T cells and their expansion to concentrations sufficient for a therapeutic dose based on previous studies of γδ T cell therapy of human xenografts in immunodeficient mice [35], and ii) the retention of anti-tumor cytotoxicity of the genetically engineered cells at concentrations of temozolomide that up-regulate tumor associated stress molecules that activate effector cell functions. Intra-cavity post-resection administration of glioma-reactive genetically engineered γδ T cells presents one of the few opportunities to deliver concentrated cellular immunotherapy directly to the site of residual malignancy at the time of maximal tumor vulnerability during high dose chemotherapy. The in vitro effectiveness of our γδ T cell-based DRI strategy provides the necessary foundation to pursue such an innovative approach to the treatment of high-grade gliomas.
